# Gingipains disrupt bone homeostasis via dual regulation of osteogenesis and osteoclastogenesis through exosomal miR-146a-5p/TRAF6 signaling

**DOI:** 10.3389/fcimb.2025.1614126

**Published:** 2025-08-12

**Authors:** Jiachen Dong, Yue Liao, Mengjun Sun, Huiwen Chen, Kecong Zhou, Huanyu Zhang, Wei Zhou, Zhongchen Song

**Affiliations:** ^1^ Department of Periodontology, Shanghai Ninth People’s Hospital, Shanghai Jiao Tong University School of Medicine, Shanghai, China; ^2^ College of Stomatology, Shanghai Jiao Tong University, Shanghai, China; ^3^ National Center for Stomatology, National Clinical Research Center for Oral Diseases, Shanghai Key Laboratory of Stomatology, Shanghai Research Institute of Stomatology, Shanghai, China

**Keywords:** gingipains, Kgp, RGP, osteoclastic differentiation, MiR-146a-5p, TRAF6

## Abstract

**Background:**

Gingipains are virulence factors released by *Porphyromonas gingivalis* that contribute to periodontal destruction by disrupting bone metabolism. This study aimed to evaluate the dual effects of gingipains on bone metabolism by examining their impact on osteogenesis and osteoclastogenesis, hypothesizing that gingipains regulate these processes via direct and exosomal pathways involving microRNA signaling.

**Methods:**

Clinical samples of gingival crevicular fluid, subgingival plaque, and gingival tissues were collected from 15 patients with stage III-IV periodontitis and 15 healthy controls. The effects of gingipains on bone marrow mesenchymal stem cells (BMSCs) and RAW264.7 macrophages were assessed using cell proliferation assays, qPCR, western blot, microarray analysis, and dual-luciferase reporter assays. A rat periodontitis model was used to validate the findings *in vivo*.

**Results:**

Periodontitis patients exhibited elevated levels of lysine- and arginine-specific gingipains, C5a, and RANKL (p < 0.05). Gingipains inhibited BMSCs proliferation and osteogenic differentiation in a dose-dependent manner while promoting osteoclastogenesis in RAW264.7 macrophages through BMSCs-derived exosomes. Gingipains reduced the levels of miR-146a-5p in exosomes, which enhanced osteoclast differentiation through the miR-146a-5p/TRAF6 signaling pathway. Animal models confirmed that gingipains aggravated alveolar bone loss, which was mitigated by miR-146a-5p overexpression.

**Conclusion:**

Gingipains disrupt bone metabolism by inhibiting BMSCs osteogenesis and promoting osteoclastogenesis through communication via exosomes. Targeting miR-146a-5p offers a potential therapeutic approach to counter gingipain-induced periodontal destruction.

## Introduction

Gingipains are cysteine proteases secreted by *Porphyromonas gingivalis* (*P. gingivalis*).They are major virulence factors in periodontal diseases, and they facilitate *P. gingivalis* survival and pathogenicity by promoting host colonization, evading the immune system, aiding tissue destruction, and acquiring nutrients (Dominy et al., 2019; Hocevar et al., 2018; Xu et al., 2020). As *P. gingivalis* infection increases owing to periodontal inflammation, gingipains play a major role in protein degradation, disrupting signaling pathways, evading the immune system, and causing tissue damage.

The gingipain family comprises lysine-specific gingipains (Kgps) and arginine-specific gingipains (RgpA/B), which disrupt antimicrobial responses (Reyes, 2021), and enhance *P. gingivalis* adherence (Shiheido-Watanabe et al., 2023), biofilm formation, and interactions with other pathogens, specifically red microbial complexes (Xu et al., 2020). Rgps degrade complement components C3, C4, and C5, which triggers complement–Toll like receptors (TLR) crosstalk and exacerbates inflammation by increasing tumor necrosis factor-α (TNF-α), interleukin (IL)-1β, and IL-6 levels. Conversely, Kgps modulate neutrophil recruitment by cleaving the C5a receptor (Xu et al., 2020). The increased antibody responses to gingipains in periodontitis patients emphasizes their potential as biomarkers for disease severity (de Vries et al., 2022), however, few clinical studies have linked gingipains to periodontal status.

Gingipains exert cytotoxic effects on fibroblasts, bone marrow mesenchymal stem cells (BMSCs), and epithelial cells, which disrupt the balance between regeneration and resorption (Kadowaki, 2021; Mo et al., 2020a). Moreover, gingipains directly inhibit BMSCs proliferation and osteogenic differentiation by degrading integrin β1 and suppressing RhoA activity, which results in osteoblast apoptosis (Qiu et al., 2018). Although RgpA inhibitors can reverse the reduction in osteogenic-related gene expression (Zhang et al., 2019), their overall effect on pathways involved in periodontal regeneration remains unclear.

Moreover, gingipains directly regulate macrophages by promoting M1 macrophage polarization through the C5a pathway (Hou et al., 2017). Gingipains from outer membrane vesicles activate inflammasomes, the NF-κB pathway, and osteoclastic differentiation through enhanced integrin β3 expression (Castillo et al., 2022), and osteoclastic differentiation through enhanced integrin β3 expression (Mo et al., 2020a). They further alter the periodontal inflammatory microenvironment through intracellular communication. In a previous study, we found that *Porphyromonas gingivalis*-LPS reduces miR-151-3P levels in BMSC-derived exosomes (BMExo). This reduction promotes the formation of osteoclasts in RANKL-induced macrophages. miRNAs, such as miR-124 and miR-218, regulate bone metabolism by inhibiting bone resorption, whereas miR-31 inhibitors reverse RANKL-induced osteoclastogenesis (Sadek et al., 2023), however, the mechanism by which gingipains affect communication between osteoblasts and osteoclasts remains unclear. In this study, we examined the dual effects of gingipains on periodontal regeneration by focusiong on their direct influence on osteoblasts and their indirect role in promoting osteoclastogenesis through exosomal pathways. Understanding these mechanisms will lead to more effective strategies to prevent gingipain-induced periodontal destruction.

## Methods

### Sample collection

All experimental protocols adhered to the ethical principles of the Declaration of Helsinki (revised in 2013). The study was approved by the Institutional Review Board of Shanghai Ninth People’s Hospital, Shanghai Jiao Tong University School of Medicine (NO.SH9H-2021-T112-1), with written informed consent obtained from all participants.

The criteria for inclusion included for the study were those who were systemically healthy and willing to participate in the study. Subjects were classified based on the Consensus report of workgroup 2 of the World Workshop on the Classification of Periodontal and Peri-Implant Diseases and Conditions (Papapanou et al., 2018). The periodontitis group comprised 15 patients with stage III-IV periodontitis and the health group included 15 periodontal healthy people. All subjects recruited between January 2022 and January 2023. Patients of the periodontitis group were selected from the Department of Periodontology, while the health group subjects were volunteers with a full-mouth plaque score ≤ 25% and a full-mouth bleeding score of ≤ 10%.

This study excluded patients following exclusion criteria: (1) who had contraindications (serious systemic diseases including uncontrolled hypertension, diabetes and serious cardiovascular disease) for the treatment of periodontitis later, (2) who had received periodontal treatment or anti-inflammatory medication regularly within the previous 3 months, (3) Who had used bone metabolism-affecting drugs (such as bisphosphonates, immunosuppressants) for a long time, (4) who had osteoporosis.

Gingival crevicular fluid (GCF) were collected from sites with probing depth ≥ 5 in the periodontitis group and from Ramfjörd teeth in the control group using sterile absorbent paper points as per Qiu et al (Qiu et al., 2024). Sample weights were recorded pre- and post-collection, with 20 strips gathered per participant. Subgingival plaque samples were obtained using Gracey curettes and stored in sterile tubes with phosphate buffer solution.

Gingival samples were obtained during modified Widman flap surgery for periodontitis patients and during crown lengthening procedures for healthy controls. All samples were stored at −80°C.

### Measurement of P. gingivalis DNA

Nested quantitative PCR was employed to quantify *P. gingivalis* DNA in two sequential steps: external and internal nested PCR.

### External nested PCR

A 20 μL reaction system included 10 μL Premix Ex Taq (Takara), 1 μL External Forward primer, 1μL External Reverse primer (Life Technologies), and 8 μL diluted subgingival plaque, involving 100 ng of total DNA. Amplification followed the protocol described by Fischer et al (Fischer et al., 2019).

### Internal nested PCR

The reaction mix comprised 5 μL SYBR Green MasterMix (Yeasen), 0.5 μL Internal Forward primer, 0.5 μL Internal Reverse primer, and 4 μL DNA diluted to 1/100. PCR was performed in a fluorescence quantitative PCR instrument (Roche) with the following protocol: pre-denaturation at 98°C for 5 mins; 98°C denaturation for 15s (40 cycles); annealing at 55°C for 15s, and extend at 72°C for 30s. Primer sequence of was provided in **Table 1**.

**Table 1:** The primers used in nested qPCR.

Primer name of nest *P. gingivalis*	Sequence
External Forward	GTGAGGTAACGGCTCACCAA
External Reverse	AATATGGCTTTTCGCCGTGC
Internal Forward	AGGCAGCTTGCCATACTGCG
Internal Reverse	ACTGTTAGCAACTACCGATGTA

### Measurement of lysine and arginine gingipains in gingiva

Gingival tissues were rinsed three times with cold PBS, minced, and treated with a lysis reagent containing protease inhibitors. Tissue homogenization was conducted at 60Hz for 70s, repeated twice. After centrifugation at 12,000 rpm for 10 min, proteins were separated by SDS-PAGE and transferred to a PVDF membrane. The membrane was blocked, incubated with primary and secondary antibodies as per previously described methods (Dong et al., 2024), and analyzed using ImageJ to quantify Kgps and Rgp expression.

### Detection of Kgps and RgpA/B activity in GCF

Fluorogenic substrates for Kgp, RgpA, and RgpB were diluted in a buffer (100 mM tris-HCl, 75 mM NaCl, 2.5 mM CaCl2, 10 mM cysteine, and 1% dimethyl sulfoxide) (Dominy et al., 2019). GCF samples and substrates (final concentration 10 μM) were added to 96-well plates, and fluorescence signals were recorded using a multifunctional ELISA reader over 120 min. Fluorescence parameters were as follows: excitation/emission at 380/460 nm for Kgp and 380/450 nm for Rgp.

### Enzyme-linked immunosorbent assay

ELISA kits were used to evaluate C5a and RANKL levels in GCF samples as per the manufacturer’s instructions (Simuwu). Optical density (OD) values were determined at 450 nm, with each assay performed in triplicate.

### Evaluation of gingipain-induced proliferation and osteogenic differentiation in BMSCs

The proliferation of BMSCs treated with gingipains was assessed using the MTT assay. BMSCs (5×10^4^/ml) were seeded in a 96-well plate for 24 h and treated with DMEM containing gingipains at concentrations of 0.75, 1.5, 3, 6, 12 and 24 μg/ml. After 1, 3, 5 and 7 days of co-culture, cells were incubated with 200 μL MTT solution for four hours, and formazan crystals were dissolved in 150 μL dimethyl sulfoxide. OD was measured at 490 nm using a microplate reader.

Cell viability was further assessed using a live/dead staining kit (Sigma) and fluorescence microscopy (Leica DMi8) after 3 days. Morphological changes in BMSCs were observed under a transmission electron microscope (TEM) after fixation, dehydration, embedding, and staining.

Osteogenic differentiation was evaluated by qRT-PCR and western blot analysis of osteogenic-related genes and proteins, including runt-related transcription factor 2 (RUNX2), BMP-2, alkaline phosphatase (ALP), and distal-less homeobox 5 (DLX5). BMSCs were cultured in osteoblast-conditioned medium with or without gingipains for 3 or 7 days. The primer sequences and antibodies were described in our previous study (Dong et al., 2024).

### Identification of BMExo and their osteoclastic effects on RAW264.7 under the stimulation of gingipains

BMExo was characterized using TEM (H-7650, Hitachi) and nanoparticle tracking analysis (NTA). Exosomal marker proteins CD 9 (proteintech, 20597-1-AP, 1:4000) and CD 81 (proteintech, 27855-1- AP, 1:2000) were examined by western blot.

BMSCs were pre-treated with gingipains (BMSC-Gin) or untreated, and exosomes were isolated from the supernatant using ultracentrifugation (Guo et al., 2021).

The cytotoxic effects of BMExo on RAW264.7 (China Center for Type Culture Collection) were determined utilizing CCK-8 assay and live/dead staining after 1, 3, and 7 days of incubation. Cells were cultured in osteoclast-conditioned medium and divided into three groups: BMExo-Gin, BMExo, and control groups (osteoclast-conditioned medium only).

ELISA was used to measure protein levels of osteoprotegerin (OPG) and RANKL in the supernatant. qRT-PCR and western blot analyzed osteoclastic genes and proteins, including cathepsin K (CTSK), NFATC1, and tartrate-resistant acid phosphatase (TRAP). TRAP-positive multinucleated cell areas were quantified using ImageJ.

### Regulation of exosomal miRNA and its osteoclastic effects on RAW264.7

To clarify the mechanism of BMSC-sourced exosomes in osteoclastic differentiation, exosomal miRNA expression was analyzed via microarray. RAW264.7 cells were divided into BMExo-Gin, BMExo, and control groups. Total RNA was extracted from exosomes according to instruction of total Exosome RNA & Protein Isolation Kit (Life Technologies), and miRNAs were profiled using Multispecies miRNA 4.0 Array (Affymetrix GeneChip). Differential expression was considered significant for fold change >1.5 (*p* < 0.05).

RAW264.7 cells were transfected with miR-146a-5p inhibitor, mimics, and inhibitor/mimics NC (ribo*FECT*™ CP Transfection Kit), respectively. The expression of miR-146a-5p was examed by qRT-PCR with primers synthesized by stem-loop method and western blot. After 3 days of transfection, osteoclastic genes and proteins expression (CTSK, NFATC1, TRAP) and TRAP staining were analyzed. Intersection analysis combined miR-146a-5p targets with osteoclast-related pathways. Dual-luciferase reporter assays confirmed miR-146a-5p binding to the 3’ UTR of TRAF6. Then, the gene and protein expression of TRAF6 was also detected by qRT-PCR and western blot. Lipofectamine 2000 (Invitrogen, USA) was used to overexpress TRAF6 in RAW264.7 cells to have further exploration on the influence of miR-146a-5p in TRAF6 signaling pathway.

### Animal study

Animal experiments were approved by the approval of the Animal Ethics Committee of the Ninth People’s Hospital Affiliated to Shanghai Jiao Tong University School of Medicine (NO. SH9H-2021-A711-1). Periodontitis models were established in 8-week-old male specified-pathogen-free (SPF) SD rats by placing 5–0 sterile silk ligatures around mandibular first molars. Rats were divided into control, silk ligature, and silk ligature + gingipains groups. Gingipains (3 mg/mL) or sterile PBS were injected thrice weekly into the gingival sulcus (Qian et al., 2021). After 8 weeks, rats were euthanized for comparison.

**Figure 1:**
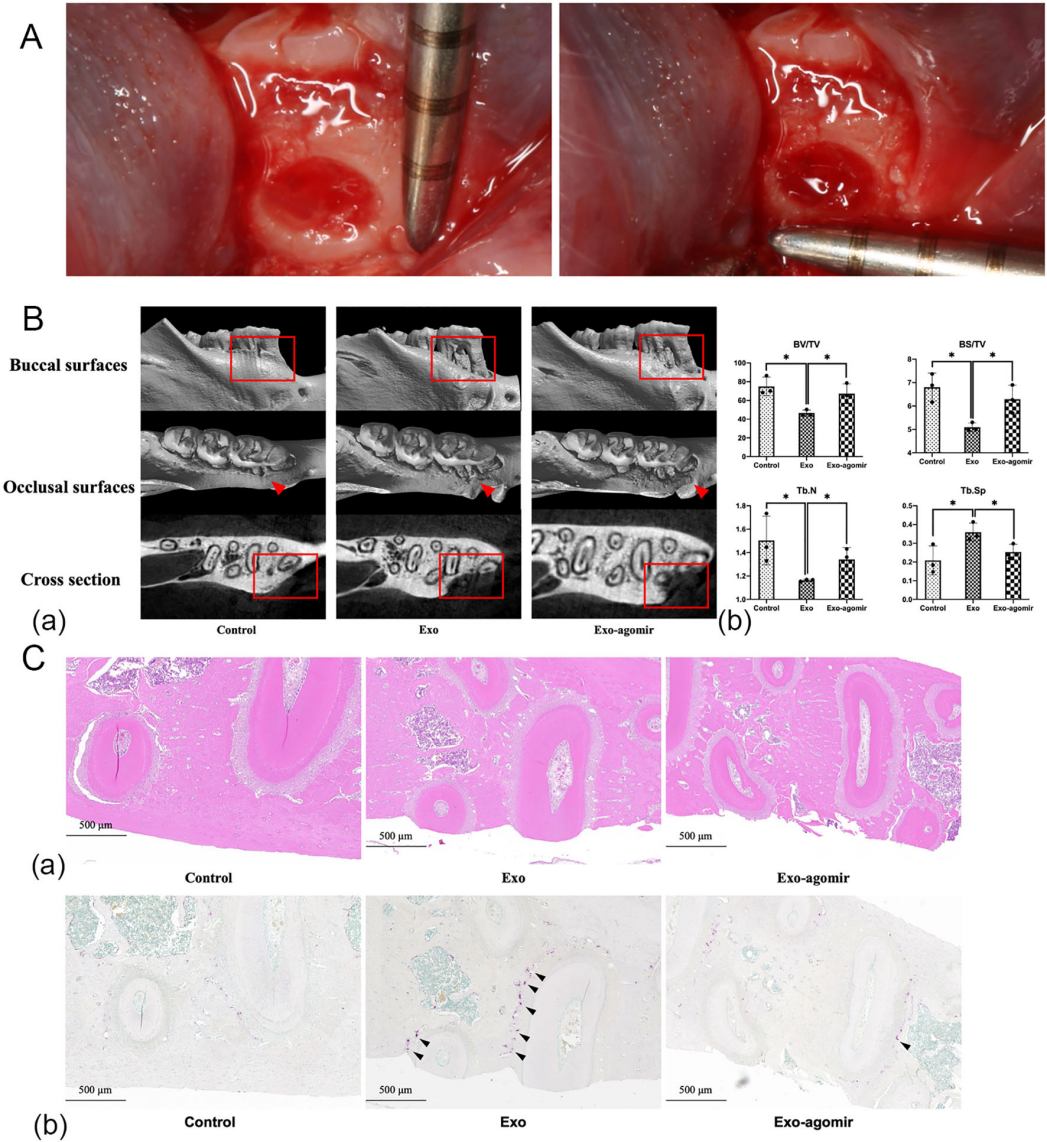
miR-146a-5p agomir promotes new bone formation in bone defect models of SD rats. **(A)** Mandibular bone defect measurement in SD rats (1 mm × 1 mm × 0.5 mm). **(B)** Reconstructed 3D microcomputed tomography images **(a)**. BV/TV, trabecular number (Tb.N), BS/BV, and Tb.Sp of mandibular bone defects **(b)** (n = 3; *P < 0.05). **(C)** Hematoxylin and eosin staining **(a)** and TRAP staining **(b)** of mandibular bone defects (scale bar: 500 μm, black arrow: osteoclasts).

For alveolar bone defect models, defects (1 mm × 1 mm × 0.5 mm) were created in mandibular first molars (**Figure 1A**). Rats were divided into control, BMExo, and BMExo-agomir groups (30 nM miR-146a-5p agomir). Exosomes and agomir were injected into alveolar bone defects every 3 days for 2 weeks (4 times in total), 200 μg exosomes and 7.5 nM miR-146a-5p agomir per time. Animals were euthanized after 8 weeks for Micro CT and histological staining.

### Statistical analysis

Statistical significance among multiple groups with one independent variable was inspected with one-way analysis of variance (ANOVA). A statistically significant difference was indicated by *p*<0.05.

## Results

### Increased expression of P. gingivalis and activities of Kgp and Rgp in periodontitis patients

**Figure 2:**
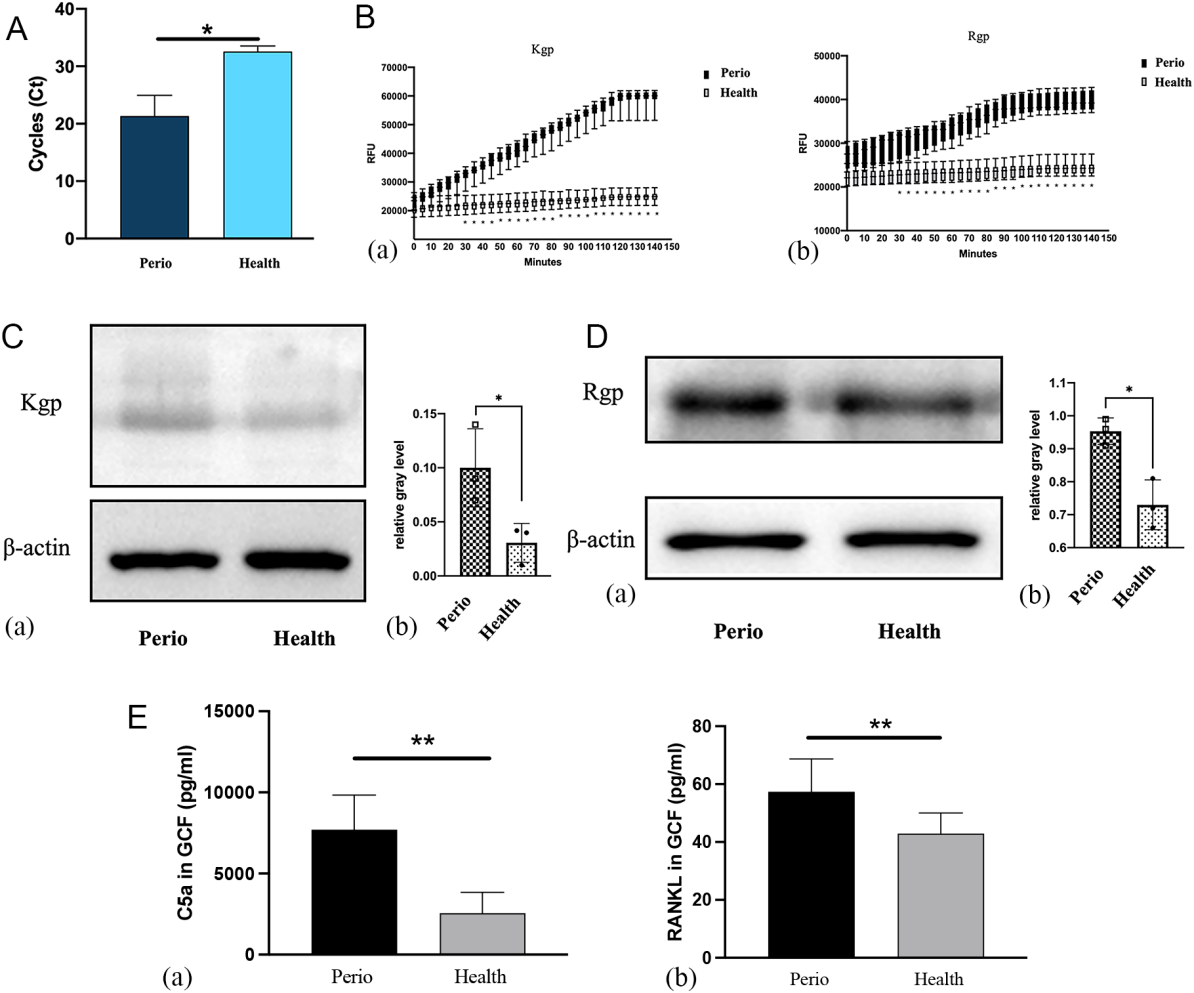
Evaluation of *Porphyromonas gingivalis* and Kgp and Rgp activities in clinical samples of patients with periodontitis vs. healthy individuals. **(A)** Detection of *P. gingivalis* in subgingival plaques detected by nested quantitative PCR (n = 15; *P < 0.05). **(B)** Kgp **(a)** and Rgp **(b)** activities were measured by ELISA (n = 15; *P < 0.05). **(C)** Kgp protein expression in the gingiva of the periodontitis group and control group **(a)**, and the relative gray levels for the two groups **(b)** (n = 3; *P < 0.05). **(D)** Rgp protein expression in the gingiva of the periodontitis group and control group **(a)**, and the relative gray levels for the two groups **(b)**. **(E)** Expression of C5a **(a)** and RANKL **(b)** protein in the GCF of the two groups (n = 15; **P < 0.01).

The relationship between gingipains and periodontium inflammation was assessed by analyzing *P. gingivalis* expression in subgingival plaque and Kgp and Rgp activities in gingiva and GCF. Nested quantitative PCR revealed significantly higher *P. gingivalis* DNA levels in patients with stage III-IV periodontitis compared to healthy controls (**Figure 2A**). Colonization with *P. gingivalis* elevated Kgp and Rgp activities, with Kgp activity in GCF significantly higher in periodontitis patients from 30 minutes onwards, peaking at 120 minutes (*p*<0.05). Rgp activity followed a similar trend, plateauing at 90 minutes (*p*<0.05; **Figure 2B**). Western blot confirmed increased Kgp and Rgp expression in gingival tissues from periodontitis patients, with Rgp levels higher than Kgp (*p*<0.05; **Figures 2C, D**). ELISA further demonstrated elevated C5a and RANKL levels in the GCF of periodontitis patients (*p*<0.05; **Figure 2E**).

### Gingipains accelerate alveolar bone resorption

**Figure 3:**
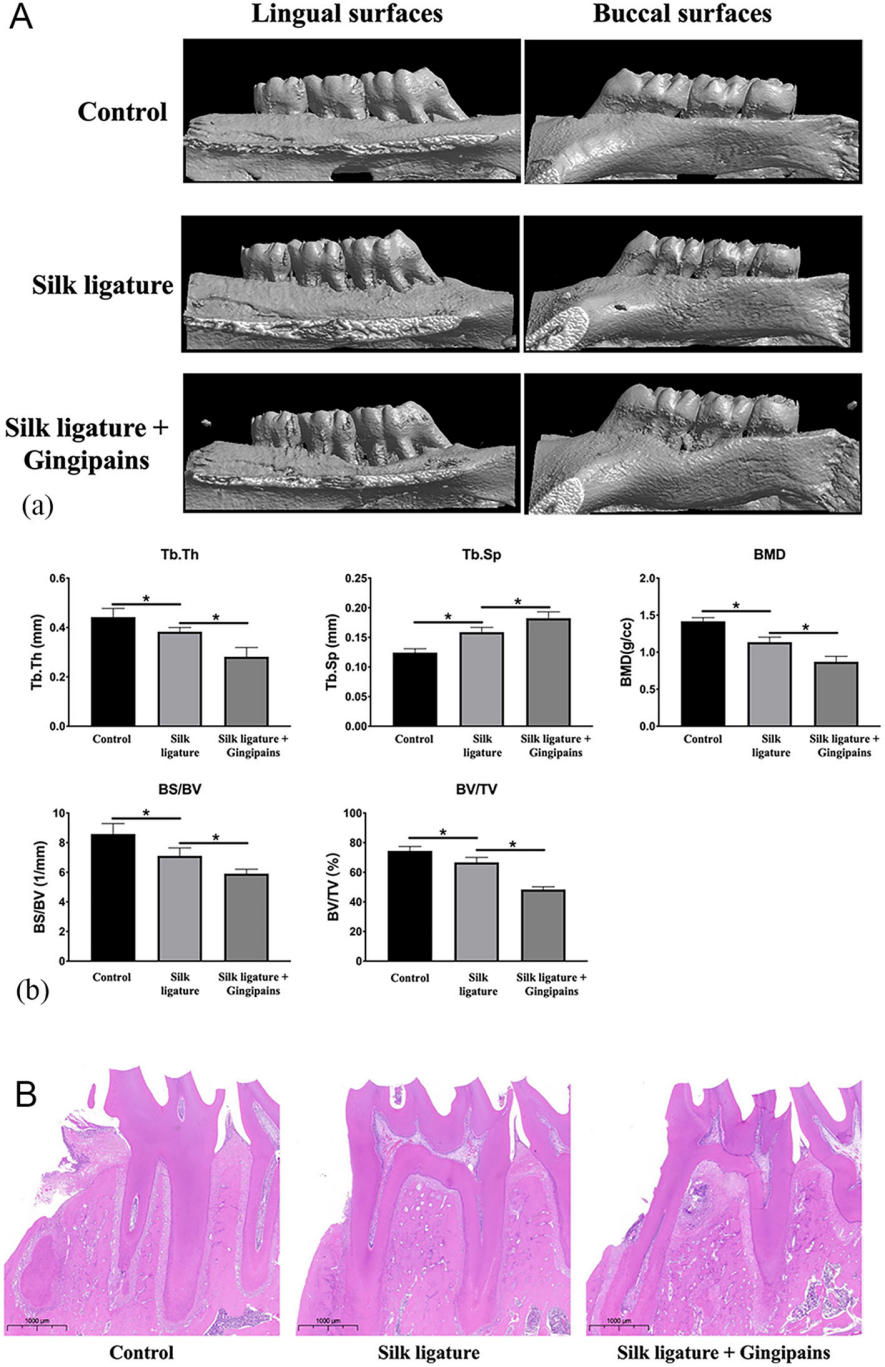
Micro-CT and HE staining of the periodontitis model in SD rats. **(A)** Reconstructed 3D microcomputed tomography images of mandibular molars and alveolar bone of the control group, silk ligature group, and silk ligature combined with the gingipains group **(a)**. Bone mineral density, trabecular thickness (Tb.Th), percent bone volume (BV/TV), trabecular separation (Tb.Sp), and bone surface/volume ratio (BS/BV) of the mandibular first molars **(b)** (n = 4; *P < 0.05). **(B)** HE staining of mandibular first molars of the three groups (scale bar: 1000 μm); *P < 0.05.

Micro-CT analysis of mandibular first molars in SD rats confirmed significant bone loss in both the 5–0 silk ligature group and the gingipains + ligature group, validating periodontitis induction (**Figure 3A-a**). Gingipains exacerbated bone loss, as evidenced by lower bone mineral density, trabecular thickness (Tb.Th), bone surface/volume ratio (BS/BV), and percent bone volume/total volume (BV/TV), along with increased trabecular separation (Tb.Sp; *p*<0.05)(**Figure 3A-b**). Hematoxylin and eosin staining showed severe destruction of alveolar bone crests in the gingipains + ligature group, with connective tissue replacing bone (**Figure 3B**).

### Inhibitory effects of gingipains on BMSCs proliferation and osteogenic differentiation

**Figure 4:**
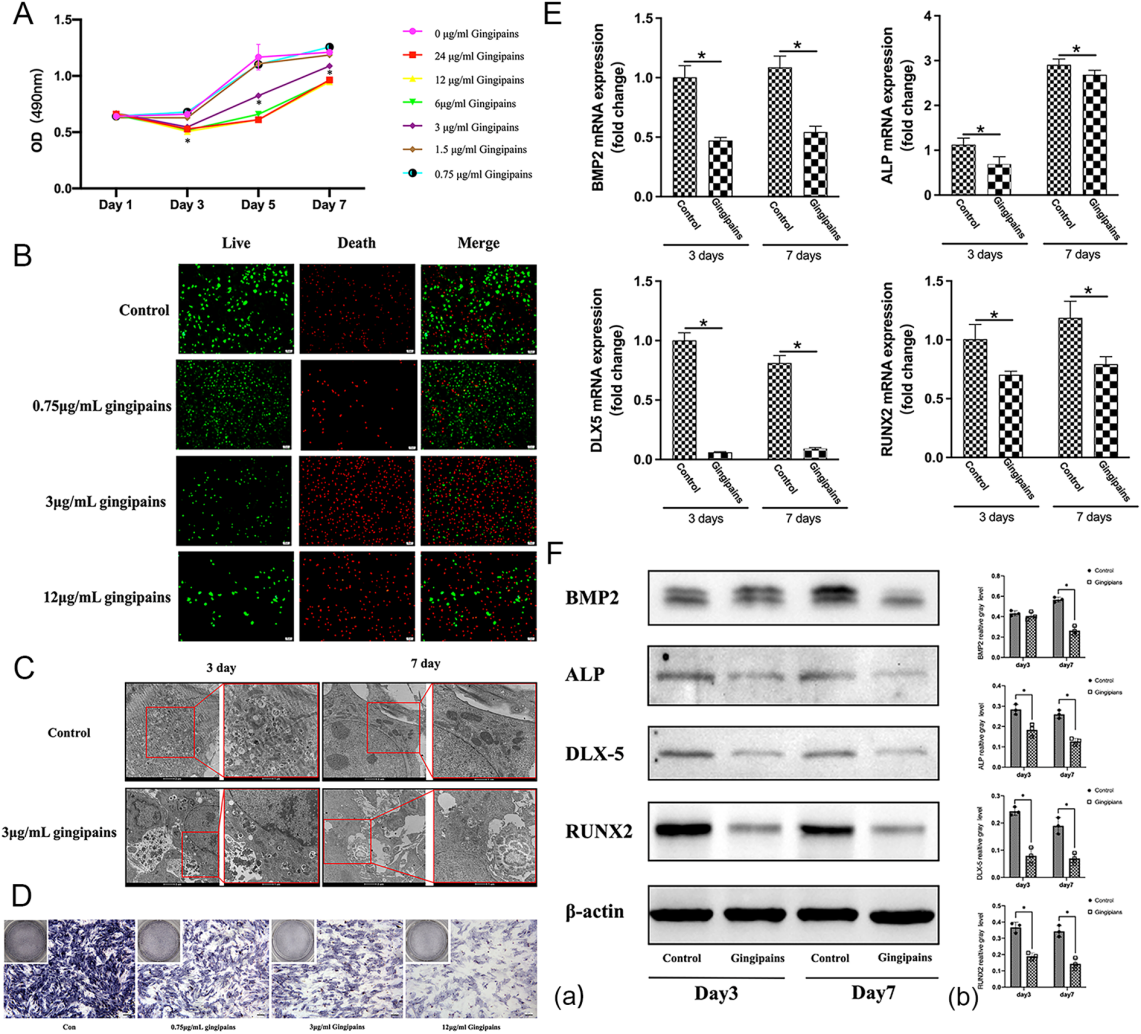
Effects of gingipain stimulation on the proliferation and osteogenic differentiation of BMSCs. **(A)** An MTT assay was used to evaluate the growth of BMSCs treated with gingipains (0–24 μg/mL) on days 1, 3, 5, and 7 (n = 5; *P < 0.05). **(B)** Live/dead staining of BMSCs under gingipains stimulation (0, 0.75, 3, and 12 μg/mL) for 3 days. **(C)** Cellular morphology of BMSCs treated with 0 or 3 μg/mL gingipains observed by transmission electron microscope (TEM). **(D)** ALP staining of BMSCs under gingipains stimulation (0, 0.75, 3, and 12 μg/mL) for 7 days. **(E)** BMP-2, ALP, distal-less homeobox 5, and runt-related transcription factor 2 mRNA expression of BMSCs treated with 3 μg/mL gingipains (n = 3; *P < 0.05). **(F)** BMP-2, ALP, distal-less homeobox 5, and runt-related transcription factor 2 protein expression of BMSCs treated with 3 μg/mL gingipains **(a)**. Relative expression of proteins was quantified by ImageJ software **(b)** (n = 3; *P < 0.05).

MTT assays revealed that gingipains at concentrations ≥3 μg/mL significantly inhibited BMSC proliferation from day 3 to day 7 (*p*<0.05; **Figure 4A**). Fluorescence microscopy showed predominantly green-stained cells in the control and 0.75 μg/mL gingipain groups, while the 3 μg/mL and 12 μg/mL groups displayed more red-stained (non-viable) cells (**Figure 4B**). TEM analysis showed intact BMSC structures in controls, but the 3 μg/mL group exhibited organelle dissolution and mitochondrial damage, indicating cytotoxicity (**Figure 4C**).

ALP staining, qPCR, and western blot results demonstrated suppressed osteogenic differentiation in gingipain-treated BMSCs. ALP activity and osteogenic-related gene expression (BMP-2, ALP, DLX5, and RUNX2) were significantly reduced in the 3 μg/mL group at days 3 and 7 (*p*<0.05; **Figures 4D–F**). BMP-2 protein expression declined significantly only on day 7 (*p*<0.05).

### BMSCs promote osteoclastic differentiation of RAW264.7 cells through exosomes stimulated by gingipains

**Figure 5:**
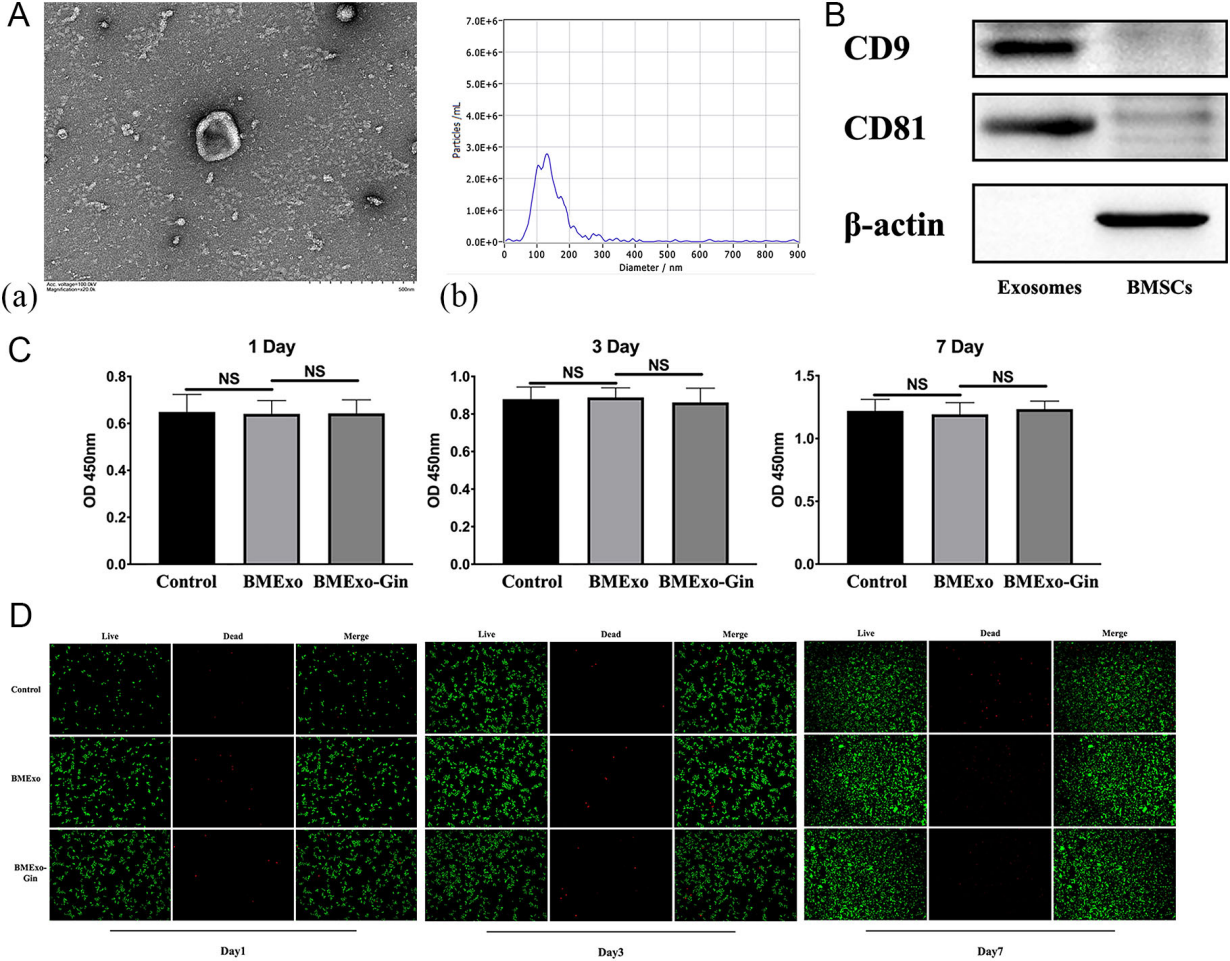
Identification and cytotoxic effect of BMSC-derived exosomes. **(A.a)** Morphology of BMSC-derived exosomes observed via TEM, and the size distribution of exosomes determined by nanoparticle tracking analysis (NTA) **(A.b)**. **(B)** The CD9 and CD81 protein levels of BMSCs and exosomes as determined by western blot analysis. **(C)** Cell viabilities of RAW264.7 co-cultured with exosomes derived from the BMExo and BMExo-Gin groups were evaluated by a CCK-8 assay on days 1, 3, and 7 (n = 10; NS, no significance). **(D)** The live/dead staining of RAW264.7 co-cultured with exosomes derived from the BMExo and BMExo-Gin groups on days 1, 3, and 7.

Scanning electron microscopy revealed that BMExo were spherical with a bilayer membrane structure (**Figure 5A.a**), and NTA measured an average exosome size of 127 nm (**Figure 5A.b**). Western blot analysis confirmed elevated expression of exosomal markers CD9 and CD81 in the exosome group (**Figure 5B**). No significant differences in OD values were observed across the control, BMExo, and BMExo-Gin groups at days 1, 3, and 7 (**Figure 5C**). Live/dead staining further demonstrated no cytotoxic effects from exosomes on RAW264.7 cells (**Figure 5D**).

**Figure 6:**
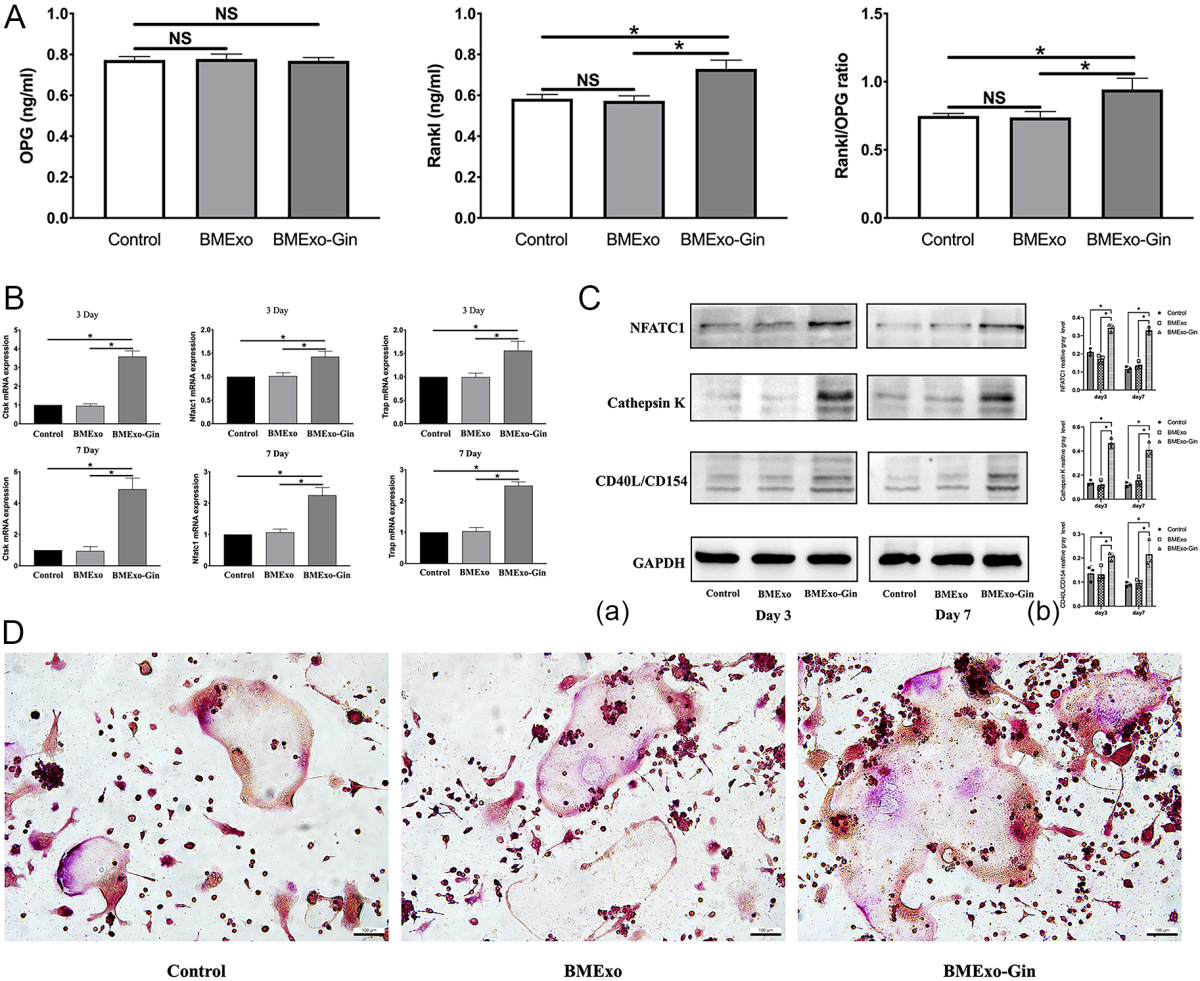
Exosomes from the BMExo-Gin group promote the osteoclastic differentiation of RAW264.7 cells. **(A)** Expression of RANKL and OPG protein in the supernatant of the control group, BMExo group, and BMExo-Gin group (n = 6; NS, no significance; *P < 0.05). **(B)** CTSK, NFATC1, and TRAP mRNA expression of RAW264.7 cells in the control, BMExo, and BMExo-Gin groups on days 3 and 7 (n = 6; *P < 0.05). **(C)** Expression of CTSK, NFATC1, and CD40L/CD154 in RAW264.7 cells on days 3 and 7 **(a)**. Relative protein expression was quantified using ImageJ software **(b)** (n = 3; *P < 0.05). **(D)** TRAP staining of RAW264.7 cells after co-culture with the exosomes of the control, BMExo, and BMExo-Gin groups for 3 days.

The BMExo-Gin group showed significantly increased RANKL protein expression and an elevated RANKL/OPG ratio compared to the control and BMExo groups, suggesting that BMExo-Gin promoted osteoclastogenesis by enhancing RANKL expression (*p*<0.05; **Figure 6A**). This was confirmed by qPCR and western blot, which showed upregulated NFATC1, CTSK, and TRAP expression in the BMExo-Gin group on days 3 and 7 (*p*<0.05; **Figures 6B, C**). Additionally, TRAP staining revealed larger TRAP-positive multinucleated cell areas in the BMExo-Gin group (**Figure 6D**).

### miR-146a-5p deficiency facilitates osteoclastic differentiation

**Figure 7:**
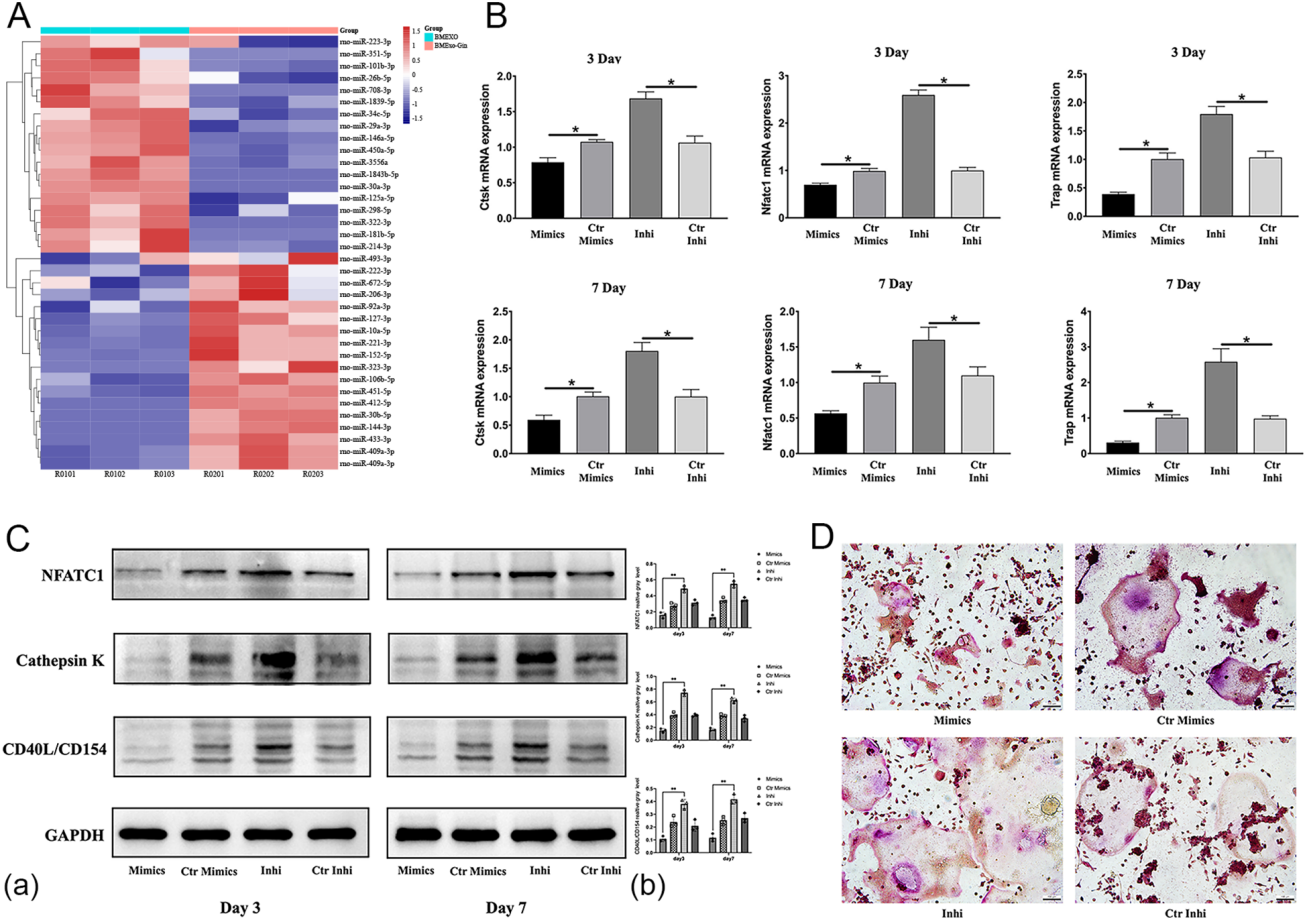
miR-146a-5p deficiency facilitates the osteoclastic differentiation of RAW264.7 cells *in vitro*. **(A)** Heat map diagram of the differential expression of exosomal miRNAs identified by miRNA microarray analysis. **(B)** Expression of osteoclastic-associated genes (*NFATC1*, *CTSK*, and *TRAP*) in RAW264.7 cells transfected with miR-146a-5p mimics (Mimic group), control mimics (Ctr Mimic group), inhibitor (Inhi group), and control inhibitor (Ctr Inhi group) (n = 6; *P < 0.05). **(C)** Expression of NFATC1, CTSK, and CD40L/CD154 protein levels in RAW264.7 cells (Mimic, Ctr Mimic, Inhi, and Ctr Inhi group) **(a)**. Relative protein expression was quantified by ImageJ software **(b)** (n = 3; *P < 0.05). **(D)** TRAP staining of RAW264.7 cells (Mimic, Ctr Mimic, Inhi, and Ctr Inhi groups). *P < 0.05.

Microarray analysis identified a significant downregulation of miR-146a-5p in BMExo from the BMExo-Gin group (**Figure 7A**). This deficiency likely contributed to enhanced osteoclastic differentiation in RAW264.7 cells treated with gingipain-stimulated exosomes.

To validate this, RAW264.7 cells were transfected with miR-146a-5p mimics or inhibitors. Inhibitor-transfected cells showed elevated expression of osteoclastic genes and proteins (NFATC1, CTSK, TRAP), while mimic-transfected cells exhibited reduced expression (**Figures 7B, C**). TRAP staining corroborated these findings, with a larger osteoclast area in the inhibitor group and a reduction in the mimic group (**Figure 7D**).

*In vivo*, miR-146a-5p deficiency exacerbated bone loss in alveolar bone defect models. Rats injected with exosomes from the BMExo-Gin group exhibited lower BV/TV, trabecular number, and BS/TV, alongside increased Tb.Sp (*p*<0.05). These effects were reversed by miR-146a-5p agomir injection (**Figure 1B**). Histological and TRAP staining revealed insufficient new bone formation and more osteoclasts in the BMExo-Gin group, further confirming miR-146a-5p’s protective role (**Figure 1C**).

### TRAF6 identified as a target gene of miR-146a-5p promoted osteoclastogenesis

**Figure 8:**
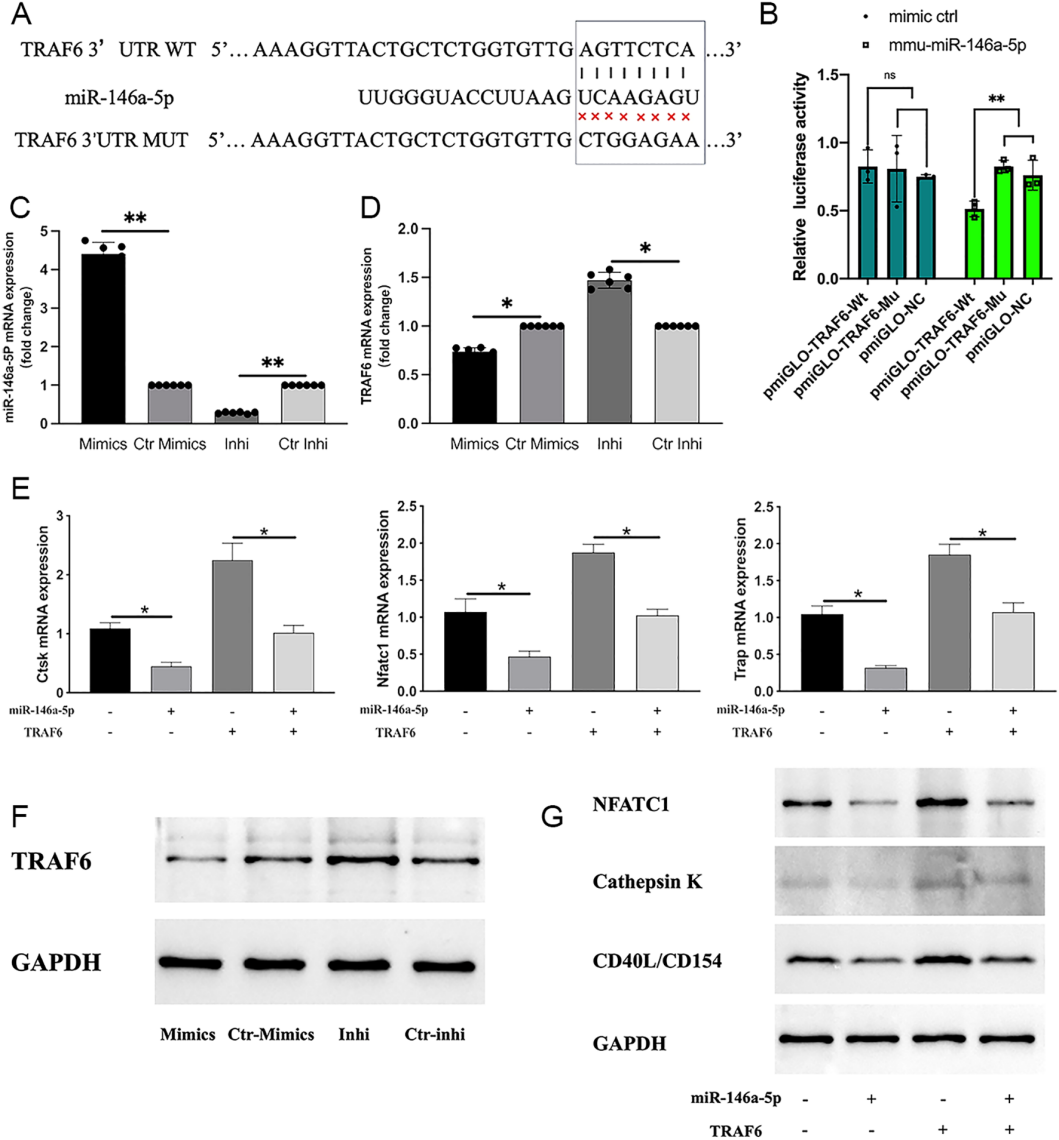
TRAF6 was identified as a downstream target of miR-146a-5p. **(A)** miR-146a-5p’s potential binding sites on the 3′ UTR of TRAF6. **(B)** Luciferase expression levels in cells transfected with miR-146a-5p determined by dual-luciferase reporter assays. (NS, no significance; **P < 0.01). **(C, F)** miR-146a-6p expression after transfection detected using qRT-PCR (n = 6; *P < 0.05) and western blot analysis (n = 3). **(D)** TRAF6 expression after transfection detected by qRT-PCR (n = 6; *P < 0.05). **(E, G)** Expression levels of osteoclastic-associated genes (n = 6; *P < 0.05) and proteins (n = 3) (NFATC1, CTSK, and TRAP) of RAW264.7 cells with or without TRAF6 and miR-146a-5p transfection.

Bioinformatic analysis identified TRAF6 as a downstream target of miR-146a-5p involved in osteoclast differentiation. A dual-luciferase reporter assay verified this mechanism. Binding sites for miR-146a-5p were present in the 3’UTR of TRAF6 (**Figure 8A**). Co-transfection with TRAF6 3’UTR wild-type plasmids and miR-146a-5p significantly reduced luciferase activity (37.78% inhibition; **Figure 8B**), whereas mutant plasmids showed no inhibitory effect. After the successful transfection of miR-146a-5p, proving by qRT-PCR (**Figure 8C**), the gene and protein expressions of TRAF6 were down-regulated in RAW 264.7 (**Figure 8D**), which revealing that miR-146a-5p negatively regulated the expression of TRAF6. What’s more, overexpression of TRAF6 up-regulated the expression of CTSK, NFATC1, TRAP, which could be reversed by overexpression of miR-146a-5p (**Figures 8E-G**).These findings confirm that exosomal miR-146a-5p regulates osteoclast differentiation in RAW264.7 cells via its downstream target TRAF6.

## Discussion

Treating severe periodontitis remains challenge owing to an inadequet understanding of how bacterial metabolites affect periodontal tissues. *P. gingivalis* is a prominent oral pathogen that contributes substantially to periodontal inflammation. Its major virulence factors, known as gingipains, play an important role in periodontal inflammation; however, the exact mechanisms by which they regulate osteogenesis and osteoclastogenesis during periodontitis remain unclear. In this study, we evaluated the correlation between gingipain expression and periodontitis severity. We also investigated how gingipains affect the osteogenic differentiation of BMSCs and their interaction with osteoclasts.

Clinical samples revealed increased levels of *P. gingivalis* DNA and increased gingipain expression in the subgingival plaque and gingival tissues of patients with stage III–IV periodontitis. Increased Kgp and Rgp activities, along with higher levels of C5a and RANKL in GCF, were positively correlated with disease severity, which suggests the involvement of gingipains in periodontal destruction. These findings highlight the advantages of targeting gingipains, as natural and synthetic inhibitors of Kgp and Rgp can ameliorate periodontal inflammation (Chow et al., 2022; Sadek et al., 2023).

Our findings are consistent with those of previous studies, such as How et al (How et al., 2016). They reported that *P. gingivalis* was detected in 87.75% of periodontitis patients, and was closely linked to disease severity. In another study, the keratin 6 fragment, a Kgp cleavage product, was identified as a marker of active gingipains in periodontitis (Tancharoen et al., 2015). This study confirmed overexpression and increased activity of gingipains in stage III–IV periodontitis, which significantly contributs to tissue destruction.

Gingipains exert significant effect on cell activity and proliferation. Zhang et al. demonstrated that gingipains stimulation increases apoptosis in human skull osteoblasts and MC3T3-E1 cells, which is marked by elevated caspase-3 levels and DNA fragmentation (Zhang et al., 2017). They attributed the apoptosis to F-actin breakage caused by integrin β1 degradation and RhoA suppression. Specifically, Kgp, is a major driver of osteoblast apoptosis (Qiu et al., 2018). Similarly, our study showed that gingipains at concentrations of 3 μg/mL or higher inhibited BMSCs proliferation after 3 days, with their effect correlating positively with concentration. Studies on the effect of gingipains on osteogenic differentiation are limited. Our results indicate that 3 μg/mL gingipains suppress osteogenic differentiation in BMSCs by downregulating osteogenic genes, proteins, and ALP activity. Furthermore, transmission electron microscope analysis revealed extensive organelle damage in gingipain-treated BMSCs, which is consistent with the apoptosis-associated nuclear deformation reported by Bhardwaj and Saraf (Bhardwaj and Saraf, 2016). These results highlight the negative effects of gingipains on periodontal regeneration through the inhibition of BMSCs proliferation and osteogenic differentiation.

Moreover, gingipains contribute to bone loss by increasing osteoclast formation, particularly in macrophages induced by RANKL (Fitzpatrick et al., 2009). They increase RANKL-induced, TRAP-positive multinucleated cells and upregulate osteoclastic genes, such as *CTSK*, *NFATC1*, and *TRAF6* (Mo et al., 2020b). Gingipain (Kgp) also degrades OPG and promotes the secretion of RANKL in osteoblasts (Mo et al., 2020b; Yasuhara et al., 2009) to further induce osteoclast differentiation. Our study confirmed that gingipain-stimulated BMExo increases the RANKL/OPG ratio in RAW264.7 cells, and promotes osteoclastogenesis without exerting cytotoxic effects, as evidenced by CCK-8 assays and live/dead staining.

Exosomes are important mediators of intercellular communication in bone metabolism. They carry proteins, lipids, and miRNAs that are involved in the regulation of osteoblast and osteoclast activity (Fan et al., 2021). We demonstrated that gingipain-stimulated exosomes (BMExo-Gin) enhanc osteoclastic gene and protein expression (*CTSK, NFATC1, TRAP*) in RAW264.7 cells, as evidenced by TRAP staining. Notably, miRNAs within exosomes attenuate osteogenic and osteoclastic differentiation (Liu et al., 2023), which suggests that exosomal miRNAs have an important role in bone metabolism and osteoclastogenesis.

We sequenced exosomes from the BMExo and BMExo-Gin groups to identify differentially expressed miRNAs. Microarray analysis revealed a significant downregulation of miR-146a-5p in the BMExo-Gin group. which was previously shown to inhibit osteoclastogenesis (Lin et al., 2019) and protect female mice against age-related bone loss (Zheng et al., 2021). Its downregulation in BMExo likely promotes the osteoclastic differentiation of macrophages. Thus, to validate the role of miR-146a-5p, RAW264.7 cells were transfected with miR-146a-5p inhibitors and mimics. MiR-146a-5p knockdown enhanced osteoclastic differentiation *in vitro*, whereas its overexpression suppressed it. Similarly, animal experiments confirmed that exosomes from the BMSC-Gin group reduced new bone formation, an effect reversed by miR-146a-5p agomir. These results suggest that miR-146a-5p overexpression may be a therapeutic strategy for treating alveolar bone defects.

Bioinformatic analyses and dual-luciferase reporter assays identified TRAF6 as a direct target of exosomal miR-146a-5p. TRAF6 regulates various pathways, such as NF-κB and JNK, which are important for osteoclastogenesis. As RANK contains three TRAF6 binding sites (Gohda et al., 2005), RANKL can activates the RANKL/RANK/TRAF6 axis and downstream c-Fos pathways (Yao et al., 2021). Shao et al. demonstrated that miR-146 induces chondrocyte apoptosis by targeting the TRAF6-mediated NF-KB signaling pathway (Shao et al., 2020). Similarly, in a study on pancreatic ductal adenocarcinoma (PDAC), *in vitro* and *in vivo* experiments confirmed that miR-146a-5p is targeted to the 3′-untranslated region (3′-UTR) of TRAF6. This interaction inhibits the growth of PDAC cells and reduces the TRAF6/NF-κB p65/P-glycoprotein pathway (Meng et al., 2020).

BMExo-Gin downregulated miR-146a-5p activates the RANKL/RANK/TRAF6 axis in RAW264.7 cells. Subsequently, NF-κB activation is enhanced, which promotes the release of pro-inflammatory factors, such as TNF-α and IL-6. However, miR-146a-5p reversed gingipain-induced osteoclastogenesis by suppressing TRAF-6. This suggests its potential as a targeted drug that may ameliorate alveolar bone defects in patients with severe periodontitis.

## Conclusion

Our study elucidated how gingipains disrupt bone metabolism by exerting dual effects on osteogenesis and osteoclastogenesis. Gingipains directly inhibited BMSC proliferation and osteogenic differentiation while indirectly promoting osteoclastic differentiation via BMExo. This process involves the miR-146a-5p/TRAF6 signaling pathway, offering a potential therapeutic target. Further research is needed to fully clarify TRAF6’s role in osteoclastogenesis.

## Data Availability

The original contributions presented in the study are included in the article/supplementary material. Further inquiries can be directed to the corresponding authors.

## References

[B1] Bhardwaj J. K. Saraf P. (2016). Transmission electron microscopic analysis of malathion-induced cytotoxicity in granulosa cells of caprine antral follicles. Ultrastruct Pathol. 40, 43–50. doi: 10.3109/01913123.2015.1088908, PMID: 26513701

[B2] Castillo Y. Castellanos J. E. Lafaurie G. I. Castillo D. M. (2022). Porphyromonas gingivalis outer membrane vesicles modulate cytokine and chemokine production by gingipain-dependent mechanisms in human macrophages. Arch. Oral. Biol. 140, 105453. doi: 10.1016/j.archoralbio.2022.105453, PMID: 35580388

[B3] Chow Y. C. Yam H. C. Gunasekaran B. Lai W. Y. Wo W. Y. Agarwal T.. (2022). Implications of porphyromonas gingivalis peptidyl arginine deiminase and gingipain r in human health and diseases. Front. Cell Infect. Microbiol. 12, 987683. doi: 10.3389/fcimb.2022.987683, PMID: 36250046 PMC9559808

[B4] de Vries C. Ruacho G. Kindstedt E. Potempa B. A. Potempa J. Klinge B.. (2022). Antibodies to porphyromonas gingivalis are increased in patients with severe periodontitis, and associate with presence of specific autoantibodies and myocardial infarction. J. Clin. Med. 11(4), 1008. doi: 10.3390/jcm11041008, PMID: 35207282 PMC8875626

[B5] Dominy S. S. Lynch C. Ermini F. Benedyk M. Marczyk A. Konradi A.. (2019). Porphyromonas gingivalis in alzheimer’s disease brains: Evidence for disease causation and treatment with small-molecule inhibitors. Sci. Adv. 5, eaau3333. doi: 10.1126/sciadv.aau3333, PMID: 30746447 PMC6357742

[B6] Dong J. C. Liao Y. Zhou W. Sun M. J. Zhang H. Y. Li Y.. (2024). Porphyromonas gingivalis lps-stimulated bmsc-derived exosome promotes osteoclastogenesis via mir-151-3p/pafah1b1. Oral. Dis. 31(1), 206–216. doi: 10.1111/odi.15031, PMID: 38923332

[B7] Fan L. Guan P. Xiao C. Wen H. Wang Q. Liu C.. (2021). Exosome-functionalized polyetheretherketone-based implant with immunomodulatory property for enhancing osseointegration. Bioact Mater. 6, 2754–2766. doi: 10.1016/j.bioactmat.2021.02.005, PMID: 33665507 PMC7897935

[B8] Fischer L. A. Bittner-Eddy P. D. Costalonga M. (2019). Fetal weight outcomes in c57bl/6j and c57bl/6ncrl mice after oral colonization with porphyromonas gingivalis. Infect. Immun. 87(10), e00280-19. doi: 10.1128/IAI.00280-19, PMID: 31331955 PMC6759314

[B9] Fitzpatrick R. E. Campbell P. D. Sivagurunathan S. Pagel C. N. Potempa J. Mackie E. J.. (2009). The gingipains from porphyromonas gingivalis do not directly induce osteoclast differentiation in primary mouse bone marrow cultures. J. Periodontal Res. 44, 565–567. doi: 10.1111/j.1600-0765.2008.01151.x, PMID: 18717779

[B10] Gohda J. Akiyama T. Koga T. Takayanagi H. Tanaka S. Inoue J. (2005). Rank-mediated amplification of traf6 signaling leads to nfatc1 induction during osteoclastogenesis. EMBO J. 24, 790–799. doi: 10.1038/sj.emboj.7600564, PMID: 15678102 PMC549623

[B11] Guo S. Gu J. Ma J. Xu R. Wu Q. Meng L.. (2021). GATA4-driven miR-206-3p signatures control orofacial bone development by regulating osteogenic and osteoclastic activity. Theranostics 11 (17), 8379–8395. doi: 10.7150/thno.58052, PMID: 34373748 PMC8344011

[B12] Hocevar K. Potempa J. Turk B. (2018). Host cell-surface proteins as substrates of gingipains, the main proteases of porphyromonas gingivalis. Biol. Chem. 399, 1353–1361. doi: 10.1515/hsz-2018-0215, PMID: 29927743

[B13] Hou Y. Yu H. Liu X. Li G. Pan J. Zheng C.. (2017). Gingipain of porphyromonas gingivalis manipulates m1 macrophage polarization through c5a pathway. In Vitro Cell Dev. Biol. Anim. 53, 593–603. doi: 10.1007/s11626-017-0164-z, PMID: 28634882

[B14] How K. Y. Song K. P. Chan K. G. (2016). Porphyromonas gingivalis: An overview of periodontopathic pathogen below the gum line. Front. Microbiol. 7, 53. doi: 10.3389/fmicb.2016.00053, PMID: 26903954 PMC4746253

[B15] Kadowaki T. (2021). Enzymatic characteristics and activities of gingipains from porphyromonas gingivalis. Methods Mol. Biol. 2210, 97–112. doi: 10.1007/978-1-0716-0939-2_10, PMID: 32815131

[B16] Lin S. H. Ho J. C. Li S. C. Chen J. F. Hsiao C. C. Lee C. H. (2019). Mir-146a-5p expression in peripheral cd14(+) monocytes from patients with psoriatic arthritis induces osteoclast activation, bone resorption, and correlates with clinical response. J. Clin. Med. 8, 110. doi: 10.3390/jcm8010110, PMID: 30658492 PMC6352034

[B17] Liu Y. Zhu Z. X. Zboinski E. K. Qiu W. Lian J. Liu S.. (2023). Long non-coding rna apdc plays important regulatory roles in metabolism of bone and adipose tissues. RNA Biol. 20, 836–846. doi: 10.1080/15476286.2023.2268489, PMID: 37953645 PMC10653663

[B18] Meng Q. Liang C. Hua J. Zhang B. Liu J. Zhang Y.. (2020). A miR-146a-5p/TRAF6/NF-kB p65 axis regulates pancreatic cancer chemoresistance: functional validation and clinical significance. Theranostics 10 (9), 3967–3979. doi: 10.7150/thno.40566, PMID: 32226532 PMC7086345

[B19] Mo W. Luo H. Wu J. Xu N. Zhang F. Qiu Q.. (2020a). Gingipains promote rankl-induced osteoclastogenesis through the enhancement of integrin beta3 in raw264.7 cells. J. Mol. Histol. 51, 147–159. doi: 10.1007/s10735-020-09865-w, PMID: 32193744

[B20] Mo W. Wu J. Qiu Q. Zhang F. Luo H. Xu N.. (2020b). Platelet-rich plasma inhibits osteoblast apoptosis and actin cytoskeleton disruption induced by gingipains through upregulating integrin beta1. Cell Biol. Int. 44, 2120–2130. doi: 10.1002/cbin.11420, PMID: 32662922

[B21] Papapanou P. N. Sanz M. Buduneli N. Dietrich T. Feres M. Fine D. H.. (2018). Periodontitis: Consensus report of workgroup 2 of the 2017 world workshop on the classification of periodontal and peri-implant diseases and conditions. J. Clin. periodontology 45 Suppl 20, S162–s170. doi: 10.1111/jcpe.12946, PMID: 29926490

[B22] Qian X. Zhang S. Duan L. Yang F. Zhang K. Yan F.. (2021). Periodontitis deteriorates cognitive function and impairs neurons and glia in a mouse model of alzheimer’s disease. J. Alzheimers Dis. 79, 1785–1800. doi: 10.3233/JAD-201007, PMID: 33459718

[B23] Qiu Q. Zhang F. Wu J. Xu N. Liang M. (2018). Gingipains disrupt f-actin and cause osteoblast apoptosis via integrin beta1. J. Periodontal Res. 53, 762–776. doi: 10.1111/jre.12563, PMID: 29777544

[B24] Qiu C. Zhou W. Shen H. Wang J. Tang R. Wang T.. (2024). Profiles of subgingival microbiomes and gingival crevicular metabolic signatures in patients with amnestic mild cognitive impairment and alzheimer’s disease. Alzheimers Res. Ther. 16, 41. doi: 10.1186/s13195-024-01402-1, PMID: 38373985 PMC10875772

[B25] Reyes L. (2021). Porphyromonas gingivalis. Trends Microbiol. 29, 376–377. doi: 10.1016/j.tim.2021.01.010, PMID: 33546976

[B26] Sadek K. M. El Moshy S. Radwan I. A. Rady D. Abbass M. M. S. El-Rashidy A. A.. (2023). Molecular basis beyond interrelated bone resorption/regeneration in periodontal diseases: A concise review. Int. J. Mol. Sci. 24(5), 4599. doi: 10.3390/ijms24054599, PMID: 36902030 PMC10003253

[B27] Shao J. Ding Z. Peng J. Zhou R. Li L. Qian Q.. (2020). Mir-146a-5p promotes il-1β-induced chondrocyte apoptosis through the traf6-mediated nf-kb pathway. Inflammation Res. 69, 619–630. doi: 10.1007/s00011-020-01346-w, PMID: 32328683

[B28] Shiheido-Watanabe Y. Maejima Y. Nakagama S. Fan Q. Tamura N. Sasano T. (2023). Porphyromonas gingivalis, a periodontal pathogen, impairs post-infarcted myocardium by inhibiting autophagosome-lysosome fusion. Int. J. Oral. Sci. 15, 42. doi: 10.1038/s41368-023-00251-2, PMID: 37723152 PMC10507114

[B29] Tancharoen S. Matsuyama T. Kawahara K. Tanaka K. Lee L. J. Machigashira M.. (2015). Cleavage of host cytokeratin-6 by lysine-specific gingipain induces gingival inflammation in periodontitis patients. PloS One 10, e0117775. doi: 10.1371/journal.pone.0117775, PMID: 25688865 PMC4331500

[B30] Xu W. Zhou W. Wang H. Liang S. (2020). Roles of porphyromonas gingivalis and its virulence factors in periodontitis. Adv. Protein Chem. Struct. Biol. 120, 45–84. doi: 10.1016/bs.apcsb.2019.12.001, PMID: 32085888 PMC8204362

[B31] Yao Z. Getting S. J. Locke I. C. (2021). Regulation of tnf-induced osteoclast differentiation. Cells 11, 132. doi: 10.3390/cells11010132, PMID: 35011694 PMC8750957

[B32] Yasuhara R. Miyamoto Y. Takami M. Imamura T. Potempa J. Yoshimura K.. (2009). Lysine-specific gingipain promotes lipopolysaccharide- and active-vitamin d3-induced osteoclast differentiation by degrading osteoprotegerin. Biochem. J. 419, 159–166. doi: 10.1042/BJ20081469, PMID: 19102726 PMC4188552

[B33] Zhang R. Yang J. Wu J. Xiao L. Miao L. Qi X. (2019). Berberine promotes osteogenic differentiation of mesenchymal stem cells with therapeutic potential in periodontal regeneration. Eur. J. Pharmacol 851, 144–150. doi: 10.1016/j.ejphar.2019.02.026, PMID: 30776366

[B34] Zhang F. Qiu Q. Song X. Chen Y. Wu J. Liang M. (2017). Signal-regulated protein kinases/protein kinase b-p53-bh3-interacting domain death agonist pathway regulates gingipain-induced apoptosis in osteoblasts. J. Periodontol. 88, e200–e210. doi: 10.1902/jop.2017.160806, PMID: 28691888

[B35] Zheng M. Tan J. Liu X. Jin F. Lai R. Wang X. (2021). Mir-146a-5p targets sirt1 to regulate bone mass. Bone Rep. 14, 101013. doi: 10.1016/j.bonr.2021.101013, PMID: 33855130 PMC8024884

